# Branch Retinal Vein Occlusion After COVID-19 Infection: A Case Report

**DOI:** 10.7759/cureus.38172

**Published:** 2023-04-26

**Authors:** Petros Kapsis, Chrysa Agapitou, Eleni Dimitriou, Panagiotis Theodossiadis, Irini Chatziralli

**Affiliations:** 1 2nd Department of Ophthalmology, School of Medicine, “Attikon” University Hospital, National and Kapodistrian University of Athens, Athens, GRC

**Keywords:** mild, aflibercept, infection, branch retinal vein occlusion, covid 19

## Abstract

A 65-year-old male patient presented to the ED complaining of blurred vision in the left eye for the last three days. The patient had just recovered from COVID-19 infection and had a negative polymerase chain reaction (PCR) test two days after the initiation of symptoms. His family and medical history were clear. Ophthalmological examination and imaging revealed branch retinal vein occlusion (BRVO) with macular edema in the left eye, while the right eye was normal. The visual acuity was 6/6 in the right eye and 6/36 in the left eye. Laboratory tests, as well as the full cardiovascular and thrombophilia evaluation, were normal. Since the patient did not have known risk factors for BRVO, we hypothesize that it was related to COVID-19 infection. However, the causality between the two entities remains under investigation.

## Introduction

An outbreak of SARS‑CoV‑2 was first reported in Wuhan, China, in December 2019 [1] and spread quickly worldwide, causing COVID-19 infection, which was declared a pandemic by the WHO [2]. Although most patients with COVID-19 have been found to be asymptomatic or have mild symptoms, the disease may lead to acute respiratory distress syndrome and, in some cases, death [3]. The most common symptoms of COVID-19 include headache, fever, cough, dyspnea, anosmia, and loss of taste [3]. At the same time, ocular manifestations have been described, such as conjunctivitis, intraocular inflammation, retinal microvascular changes, and retinal vascular occlusions [4]. Herein, we present the case of branch retinal vein occlusion (BRVO) in a male patient without known risk factors, who had recently recovered from COVID-19 infection before the BRVO development.

## Case presentation

**Figure 1:**
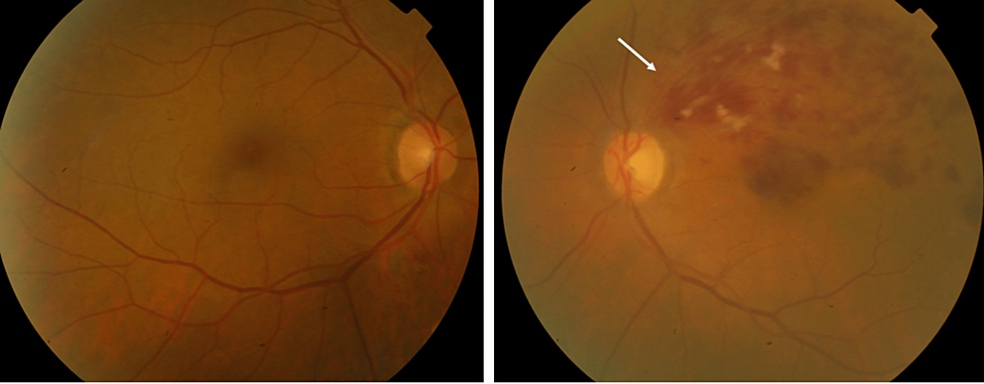
Color fundus photograph of the right eye (left panel) and the left eye (right panel), showing branch retinal vein occlusion in the left eye (white arrow).

A 65-year-old male patient presented at the ED complaining of blurred vision for the last three days. His medical and family history was clear. He was not a smoker, and he did not use any medications. He only mentioned that he had recovered from COVID-19 two days before the initiation of ocular symptoms, with a negative polymerase reaction chain (PCR) test. It is worth mentioning that during the COVID-19 infection, which was confirmed with a positive PCR test, he presented moderate symptoms with high fever and intense cough, but there was no need for hospitalization. At the presentation, a comprehensive ophthalmic examination was performed. Best-corrected visual acuity was 6/6 in the right eye and 6/36 in the left eye. Pupils were reactive to light bilaterally. Ocular motility was normal, without diplopia. The anterior segment on the slit-lamp examination was normal. Intraocular pressure was 14 mmHg in the right eye and 15 mmHg in the left eye. Dilated fundoscopy revealed tortuous and dilated retinal veins with flame-shaped hemorrhages, cotton-wool spots, and exudates that expanded to the superior temporal arcade, including the macula, consistent with BRVO (Figure 1).

**Figure 2:**
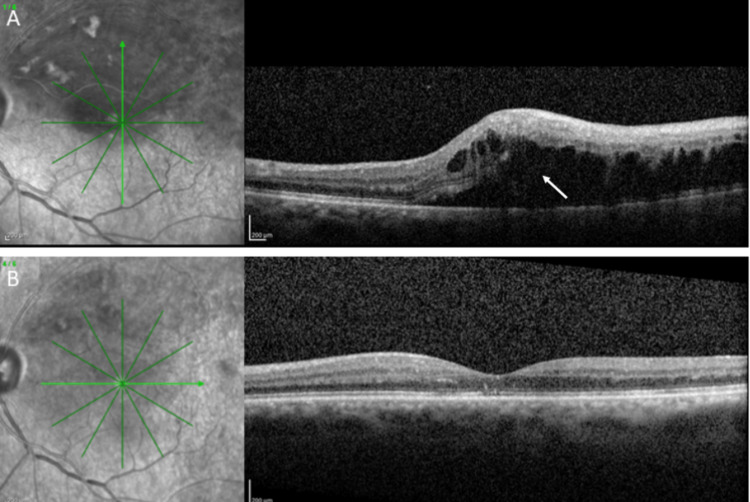
Infrared photograph and optical coherence tomography at baseline (A) showing macular edema (white arrow) due to branch retinal vein occlusion at the upper temporal retinal vein with disruption of the external limiting membrane and the ellipsoid zone, as well as the presence of intraretinal cysts in the left eye, and one month after one intravitreal aflibercept injection (B), showing resolution of macular edema.

**Figure 3:**
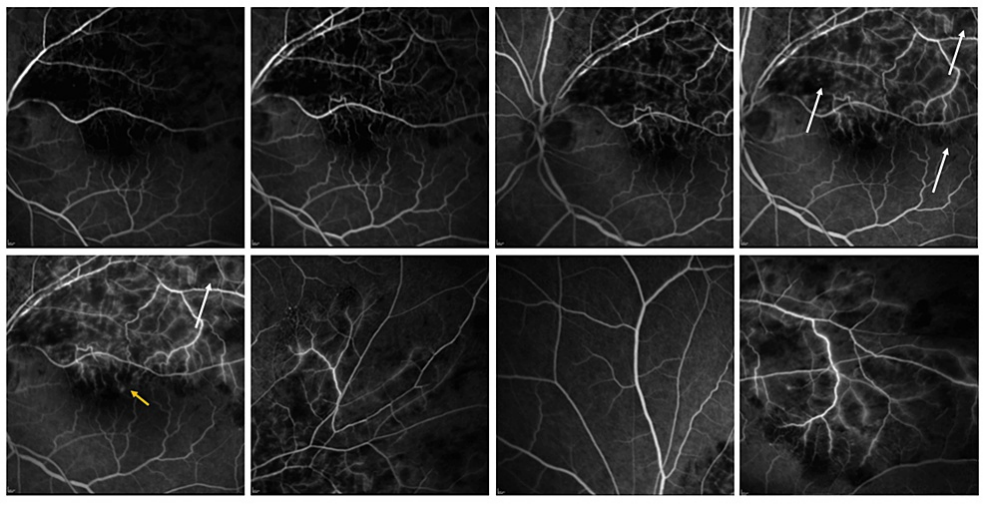
Fluorescein angiography showing hypofluorescent areas (white arrows) due to masking of secondary to retinal hemorrhages at the upper temporal quadrant, as well as hyperfluorescence (leakage at the late phase) at the macular area (orange arrow) due to macular edema, consistent with upper temporal retinal vein occlusion.

Optical coherence tomography (OCT) showed cystoid macular edema (Figure 2A). At the same time, fluorescein angiography confirmed the clinical diagnosis, depicting multiple areas of hypofluorescence due to retinal hemorrhages and areas of hyperfluorescence at the macula due to leakage secondary to macular edema (Figure 3). There were no signs of extensive ischemia at the macular area or the periphery. 

The cardiological examination and laboratory tests for dyslipidemia (total cholesterol, low-density lipoprotein [LDL], high-density lipoprotein [HDL], triglycerides, lipoprotein-a, apolipoprotein-A, and apolipoprotein-B) were normal. Blood pressure was measured to be 121/72 mmHg. In addition, the erythrocyte sedimentation rate was 5 mm/hr, and fasting blood glucose was 101 mg/dl. Prothrombin time (11.5 sec), activated partial thromboplastin clotting time (25 sec), and international normalized ratio (INR: 1.1) were all normal. A hypercoagulability panel, including full blood count, plasma homocysteine level, antiphospholipid antibody panel, protein S, protein C levels, and D-dimers, was also sent and was normal. The patient was treated with one intravitreal aflibercept injection (2.0 mg/0.05 ml), and macular edema was resolved with significant improvement in the visual acuity in the left eye from 6/36 at baseline to 6/9 one month after injection (Figure 2B). Informed consent was obtained from the patient to publish his data.

## Discussion

Retinal vein occlusion is the second most common retinal vascular disorder after diabetic retinopathy, with a global prevalence to be reported about 0.77% in people aged 30-89 [5]. The most prominent risk factors for BRVO development are hypertension and age, but other risk factors have also been identified, including diabetes mellitus, dyslipidemia, thrombophilia, and systemic and inflammatory diseases [6]. The pathogenesis of BRVO is thought to follow the principles of Virchow’s triad for thrombogenesis, i.e., damage to the vessel wall, venous stasis, and hypercoagulability [6]. In the COVID-19 era, there has been reported an increase in the incidence of RVO cases after COVID-19, although these events remain rare, and a cause-and-effect relationship cannot be established [7]. In fact, the pathogenesis of COVID-19 remains elusive. However, there is evidence that inflammation with cytokine production and consequent activation of the coagulation cascade and a cellular immune response may play a crucial role in disease progression [8]. COVID-19-related inflammation may cause platelet activation, endothelial dysfunction, and stasis, predisposing patients to thromboembolic events in the arterial and venous circulation [9,10]. Based on the above, retinal vascular damage in COVID-19 disease may be attributed to two major potential mechanisms; first, it has been reported that a viral infiltration of the endothelial cells may lead to a pseudo-vasculitis state, and second a hypercoagulable state may cause a disseminated intravascular coagulation-like scenario [10-12]. Previously, cases have been reported with BRVO after COVID-19 [10,12-19]. The significance of COVID-19 as a risk factor for systemic venous thrombosis has been well-documented among patients hospitalized for hypoxemia or other systemic complications of COVID-19 infection. However, whether patients with mild symptoms or even asymptomatic patients present similar complications remains unclear [13]. In our case, the patient had mild symptoms of COVID-19, and a positive PCR test confirmed the diagnosis. In addition, during the work-up, our patient did not have any significant medical history, and all laboratory tests and cardiovascular examinations were normal. Our patient was treated with one intravitreal aflibercept injection and showed total resolution of macular edema, suggesting that the prognosis seems to be good in such cases of BRVO, as was shown in a case series by Shiroma HF et al. [10].

## Conclusions

Since our patient with unilateral BRVO, shortly after recovery from COVID-19, was healthy without remarkable medical history and any identifiable risk factors, we hypothesize that BRVO in this patient may be due to the thromboembolic state associated with SARS-CoV-2 infection. However, the causality remains to be elucidated.
